# Vitamin D: A Role Also in Long COVID-19?

**DOI:** 10.3390/nu14081625

**Published:** 2022-04-13

**Authors:** Luigi Barrea, Ludovica Verde, William B. Grant, Evelyn Frias-Toral, Gerardo Sarno, Claudia Vetrani, Florencia Ceriani, Eloisa Garcia-Velasquez, José Contreras-Briceño, Silvia Savastano, Annamaria Colao, Giovanna Muscogiuri

**Affiliations:** 1 Dipartimento di Scienze Umanistiche, Università Telematica Pegaso, Via Porzio, Centro Direzionale, Isola F2, 80143 Napoli, Italy; luigi.barrea@unina.it; 2 Centro Italiano per la Cura e il Benessere del Paziente con Obesità (C.I.B.O), Endocrinology Unit, Department of Clinical Medicine and Surgery, University Medical School of Naples, Via Sergio Pansini 5, 80131 Naples, Italy; lud.verde@studenti.unina.it (L.V.); ambulatoriocibo@gmail.com (S.S.); operafederico2@gmail.com (A.C.); 3 Sunlight, Nutrition, and Health Research Center, P.O. Box 641603, San Francisco, CA 94164-1603, USA; wbgrant@infionline.net; 4 School of Medicine, Universidad Católica Santiago de Guayaquil, Av. Pdte. Carlos Julio Arosemena Tola, Guayaquil 090615, Ecuador; evelyn.frias@cu.ucsg.edu.ec; 5 General Surgery and Kidney Transplantation Unit, San Giovanni di Dio e Ruggi d’Aragona University Hospital, 84131 Salerno, Italy; gsarno79@yahoo.it; 6 Unit of Endocrinology, Dipartimento di Medicina Clinica e Chirurgia, Federico II University Medical School of Naples, Via Sergio Pansini 5, 80131 Naples, Italy; c.vetrani@libero.it; 7 Nutrition School, Universidad de la Republica (UdelaR), Montevideo 11100, Uruguay; florceriani@gmail.com; 8 Clinical Nutrition Service, Grupo Hospitalario Kennedy, Guayaquil 090615, Ecuador; eloisa.garciavelasquez@gmail.com; 9 Nursing Program, Universidad Técnica Particular de Loja, San Cayetano Alto, Calle París, Loja 110107, Ecuador; jocontreras@utpl.edu.ec; 10 Cattedra Unesco “Educazione Alla Salute e Allo Sviluppo Sostenibile”, Federico II University, 80131 Naples, Italy

**Keywords:** COVID-19, SARS-CoV-2, long COVID-19, vitamin D, inflammation

## Abstract

Coronavirus disease 2019 (COVID-19) has quickly become a global pandemic. Reports from different parts of the world indicate that a significant proportion of people who have recovered from COVID-19 are suffering from various health problems collectively referred to as “long COVID-19”. Common symptoms include fatigue, shortness of breath, cough, joint pain, chest pain, muscle aches, headaches, and so on. Vitamin D is an immunomodulatory hormone with proven efficacy against various upper respiratory tract infections. Vitamin D can inhibit hyperinflammatory reactions and accelerate the healing process in the affected areas, especially in lung tissue. Moreover, vitamin D deficiency has been associated with the severity and mortality of COVID-19 cases, with a high prevalence of hypovitaminosis D found in patients with COVID-19 and acute respiratory failure. Thus, there are promising reasons to promote research into the effects of vitamin D supplementation in COVID-19 patients. However, no studies to date have found that vitamin D affects post-COVID-19 symptoms or biomarkers. Based on this scenario, this review aims to provide an up-to-date overview of the potential role of vitamin D in long COVID-19 and of the current literature on this topic.

## 1. Introduction

Because of the recent advances in the pathophysiological mechanisms that occur with the novel coronavirus disease 2019 (COVID-19), there is growing interest to profoundly investigate the role of vitamin D and its deficiency to increase the susceptibility and negative results of COVID-19. Vitamin D is a secosteroid produced by the skin mainly due to exposure to sunlight in the form of cholecalciferol, and diet provides about 20% of the daily requirement of this vitamin [1].

Vitamin D deficiency has been defined as a serum concentration of 25-hydroxyvitamin D (25OHD) < 20 ng/mL (50 nmol/L) [2]. It is noteworthy to emphasize that it has been reported that its concentrations in women are lower than in men due to the proportion and distribution of fat tissue [3].

Goërtz et al. assessed 2113 patients with confirmed or suspected COVID-19 diagnosis and found that at least 87% of them continued with symptoms later than 60 days of the first symptom (of which 32% reported one or two symptoms and 55% reported three or more symptoms) [4]. The conditions (up to 60 days) following infection with COVID-19 are known as prolonged, long-lasting, post-acute, long-term, or chronic effects; among them, the most common symptoms are fatigue, dyspnea, and insomnia [5]. Vitamin D deficiency has also been related to all these symptoms [6]. Carpagnano et al. found a high prevalence of vitamin D deficiency in COVID-19 patients with acute respiratory failure [7]. It is important to emphasize that vitamin D deficiency is related to many other diseases and conditions that will increase the risk of developing a long-term COVID-19 [2,4,6,8]. In this respect, Savanelli et al. reported that vitamin D deficiency is the greatest predictor of the prevalence of dyslipidemia and hypertension in patients with coronary heart disease, suggesting the presence of both factors in cardiovascular risk in this group of patients [8]. Furthermore, according to a recent review, this virus may provoke a new onset of type 2 diabetes mellitus with undetermined clinical and metabolic components, providing a possible role for COVID-19 in developing type 2 diabetes mellitus [9].

For these reasons, vitamin D has been identified as one of the critical components for treating COVID-19 infection [6,10,11]. However, there is more to explain of how vitamin D works in prolonged COVID-19 patients. This review aims to provide a state of the art of vitamin D’s role in long COVID-19 and the updated literature on this topic. The authors will present the impact of vitamin D deficiency on COVID-19 patients and those with long-lasting COVID-19.

## 2. Methods

In this review, data were summarized using a narrative approach, based on clinical expertise in interpretation of available evidence in the peer-reviewed journal literature. Both https://pubmed.ncbi.nlm.nih.gov/ (accessed on 15 September 2021) and https://scholar.google.com/ (accessed on 15 September 2021) were searched. Search terms included COVID-19, long COVID-19, mechanisms, risk, SARS-CoV-2, symptoms, and vitamin D. In addition, references regarding virus downregulation of vitamin D receptors were found at https://vitamindwiki.com/ (accessed on 15 September 2021). All studies evaluating vitamin D and long-COVID-19 were taken into consideration. However, the review only included (i) manuscripts in English; (ii) original articles; and (iii) prospective or retrospective observational (analytical or descriptive), experimental, or quasi-experimental studies. Non-original studies, including editorials and letters to the editor, were excluded except for one editorial that had a figure showing how vitamin D reduces risk of COVID-19. No limitations on the date of publication were imposed.

## 3. COVID-19 and Vitamin D

The world is still experiencing the pandemic of COVID-19 and its impacts. This pandemic has posed an immense threat to humans, and it is responsible for causing considerable morbidity and mortality worldwide [12]. At the pandemic’s beginning, people needed to quarantine and change their lifestyle habits, such as food ingestion or exercise, and working from home became the new routine. All these modifications are associated with less time spent outside and, as a result, less sun exposure and reduced production of vitamin D [13]. 

Vitamin D deficiency is a known public worldwide health problem affecting over a billion people [14], and its consequences cannot be underestimated. Solid evidence in these subjects proposes that vitamin D has several roles besides bone and calcium metabolism [15]. Vitamin D deficiency has been related to a variety of diseases that involve infectious diseases, preeclampsia, cancers, dental caries and periodontitis, autoimmune disorders, cardiovascular disease (CVD), chronic inflammation, type 1 and 2 diabetes mellitus, and neurological disorders that significantly increases the risk of death from respiratory tract infections in otherwise healthy individuals [2,8,16,17,18,19,20,21]. Additionally, it has been reported that vitamin D deficiency in particular patient groups, such as those in intensive care units and kidney transplant recipients, had increased adverse outcomes and mortality rates [22].

Although having diseases such as those mentioned above is often considered to indicate increased risk of COVID-19, it is more likely that vitamin D deficiency accompanying the disease is the important factor, not the disease itself. An article on risk factors for COVID-19 on in-hospital mortality rates involving 66,646 inpatients in the U.S. included analyses with respect to comorbid diseases [23]. Interestingly, rates for patients with diabetes and hypertension were much higher for those with complicated disease than not, 25.2% vs. 16.5% for complicated diabetes and 28.9 vs. 14.8% for complicated hypertension. The differences for non-complicated diabetes and hypertension were not significant.

In clinical studies, low 25OHD concentrations were associated with acute respiratory tract infections, including influenza, and increased risk of community-acquired pneumonia [12]. Some retrospective studies have shown the association of 25OHD concentrations with the severity and mortality of COVID-19 cases [24].

Vitamin D has many mechanisms by which it can lower the risk of microbial infection and death. These mechanisms can be grouped into the physical barrier, natural cellular immunity, and adaptive immunity [25,26]. It is well-established that vitamin D defends the respiratory tract by maintaining strong junctions, exterminating covered viruses by cathelicidin and defensins, and lessening the generation of proinflammatory cytokines by the innate immune system that can reduce viral replication rates and diminish concentrations of proinflammatory cytokines. Therefore, vitamin D lowers the risk of a cytokine storm that can lead to pneumonia [10]. The vitamin D immunomodulation role can improve innate immunity through the secretion of antiviral peptides, which enhance mucosal resistance, influencing both tumor necrosis factor (TNF)-α and interferon-γ [12].

As mentioned before, vitamin D enhances innate cellular immunity by the induction of antimicrobial peptides, such as cathelicidin and defensins. Cathelicidins exhibit a direct antimicrobial activity against Gram-positive and Gram-negative bacteria, viruses, and fungi. The induction of cathelicidin and defensins can obstruct viral entrance into cells and repress viral replication [27]. The innate immune system generates both proinflammatory and anti-inflammatory cytokines in response to viral and bacterial infections, as observed in COVID-19 patients [28]. Vitamin D can also help reduce the production of proinflammatory Th1 cytokines, such as TNF-α and interferon-γ. Additionally, it decreases proinflammatory cytokines production and stimulates macrophages to generate anti-inflammatory cytokines [26].

Vitamin D stimulates autophagy by intensifying the expression of the light chain 3-autophagy marker. It is essential to mention that these actions are closely linked to apoptosis, which may aid viral replication. Therefore, vitamin D may be directly related to an adequate balance between autophagy and apoptosis to maximize these antiviral responses to infection [27].

**Figure 1:**
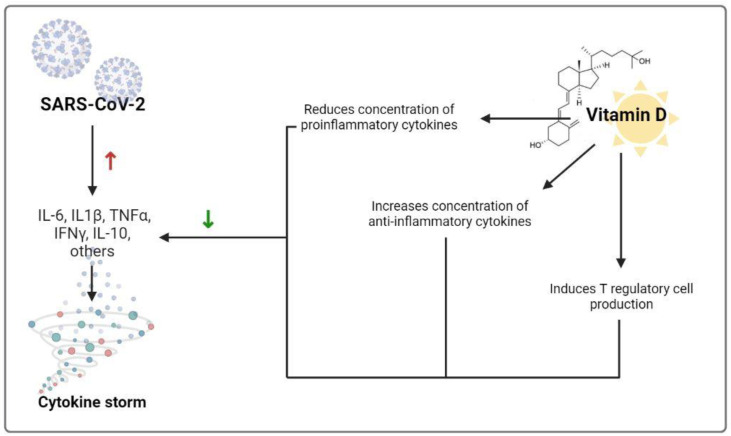
Mechanisms by which vitamin D could decrease the risk of cytokine storm. Red upward arrow indicates an increase while the green downward arrow indicates a decrease. Abbreviations: IL-6 = Interleukin-6; IL-1β = Interleukin-1β; TNFα = tumor necrosis factor α; INFγ = interferon γ; IL-10 = Interleukin-10.

It seems that severe acute respiratory syndrome coronavirus 2 (SARS-CoV-2) primarily uses the immune process during infection. It is accompanied by the release of many proinflammatory cytokines such as TNF-α, interleukin (IL)-6, and IL-1β, which are related to vascular hyperpermeability, lung injury, multiorgan failure, and COVID-19 severity, followed by hyper-reaction and cytokine storm in some patients, which can develop into a pathogenic process of acute respiratory disease syndrome [12]. The cytokine storm can lead to unusual activation of the adaptive immune pathway resulting from the alteration and disruption of the innate immune system, with proinflammatory cytokines and chemokines overflow. So, the improvement of immunity through better nutrition can be a substantial and vital factor to consider, and vitamin D shows a significant role in immune function [12] (Figure 1).

**Figure 2:**
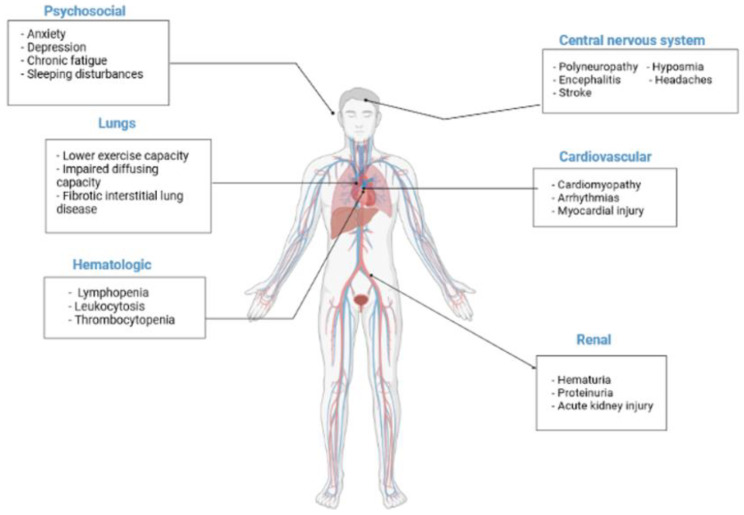
Potential long-term health consequences COVID-19. Some of the potential long-term manifestations are matched exercise capacity and carbon monoxide diffusing capacity, cardiovascular disorders, hematological manifestations and thrombotic complications, central nervous system, and psychosocial manifestations, as well as renal problems.

COVID-19 has also been associated with cardiovascular sequelae. Myocardial injury has been reported, along with the elevation of cardiac biomarkers and electrocardiographic or echocardiographic changes, cardiomyopathy, arrhythmias, thrombotic complications, and cardiogenic shock [29] (Figure 2). Activation of the vitamin D receptor also modulates myocardial contractility, likely by regulating calcium flux and low 25OHD concentrations, indicating an increased risk of overall CVD and cardiovascular mortality [30].

The large number of COVID-19 cases from the last winter is related to regular reports of the lowest 25OHD concentrations, whereas the number of cases diminished at the end of summer. These findings demonstrated that vitamin D’s protective role in decreasing the risk of acquiring COVID-19 is associated with a seasonal condition. In addition, ultraviolet-A radiation induces the release of cutaneous photolabile nitric oxide (NO), which inhibits the replication of SARS-CoV2. Furthermore, NO impacts metabolic syndrome and CVD, both COVID-19 risk factors. Cherrie et al. demonstrated the relationship between ambient ultraviolet-A radiation and COVID-19 deaths (*n* = 62,219) across the USA. The authors concluded that lower COVID-19-specific mortality is associated with higher ambient ultraviolet-A radiation exposure [31]. In addition, case-fatality incidences grow with age and chronic conditions, both of which have been related to vitamin D deficiency [26,32].

The interplay between SARS-CoV-2 and angiotensin-converting enzyme angiotensin-converting enzyme 2 (ACE2) [33] is crucial for expressing diverse clinical and metabolic characteristics. ACE2 is the host receptor to access alveolar and intestinal epithelial cells [9]. It is known that vitamin D deficiency promotes the renin-angiotensin system, leading to chronic CVD and impaired lung function [12]. The expression of the ACE2 in different tissues is proportional to various symptoms of COVID-19, such as respiratory symptom issues, acute cardiac and kidney injuries, gastrointestinal and liver function abnormalities, and beta-cell damage [9].

It has been suggested that vitamin D deficiency and lack of vitamin D receptor (VDR) activation can aggravate this respiratory syndrome associated with SARS-CoV-2, as it triggers a wounding response in lung stellate cells [34]. It is essential to point out that multiple studies identify vitamin D as capable of repairing epithelial layers and damaged organs, and the use of this vitamin in various pathologies that induce inflammation [35], such as fibrosis, appears to show that vitamin D has antifibrotic properties [36,37,38]. At the same time, a 2021 review analyzed previous animal studies where it was demonstrated that vitamin D deficiency deepened the activation of the renin−angiotensin system and increased the TGF-β/SMAD signaling pathway, thus causing bleomycin-induced lung fibrosis. The same review associated vitamin D deficiency with an increased risk of pulmonary viral infection, since it may enrich type I interferon responses, critical actors of antiviral immunity [39]. Likewise, a remarkable link has been seen between the signaling of vitamin D, the VDR, and tissue barriers. These interactions are essential in the pathogenesis of various diseases such as cancer, atopic dermatitis, and inflammatory bowel diseases [40]. It is worth highlighting the importance of this vitamin in triggering the transforming growth factor (TGF)-β-signaling pathway, essential for the proper healing of skin wounds [41]. In this respect, similar results were obtained when analyzing the effects of VDR deactivation on wound healing in mouse corneas, showing that, if VDR is inactive, this affects the healing of the corneal epithelium [42].

In many studies, the immunomodulatory characteristics of vitamin D have been reported, as well as its significant role in the maintenance of the immune system correct homeostasis; well-designed randomized controlled trials are required to discover this fascinating and plausible role of vitamin D in protective immune responses against respiratory microbes and in preventing various types of acute respiratory tract infections including SARS-CoV-2 [12]. Despite the controversy on the effect of vitamin D status on COVID-19 infection, the authors believe that vitamin D deficiency is a modifiable risk factor of acute respiratory tract infections, so it has to be considered an inexpensive, safe, and readily-available supplement for these patients [43].

Many mechanisms have been proposed to explain how vitamin D reduces the risk of COVID-19 [27,44]. Table 1 presents a list of mechanisms proposed to explain how vitamin D can reduce the risk of COVID-19. Figures showing how the vitamin D mechanisms reduce the risk of COVID-19 can be found in several publications, e.g., [27,44,45,46].

A recent meta-analysis with 23 studies (*n* = 2692) that evaluated the effect of vitamin D concentrations in COVID-19 patients reported that its deficiency appears to be related to an increased severity and mortality, but those studies did not indicate causality [61]. These determinations highlight the need for more randomized controlled trials to reach more solid conclusions.

On the other hand, an observational study based on 4.6 million community inhabitants supplemented with prescription cholecalciferol or calcifediol prescriptions in Barcelona who achieved 25OHD concentrations >30 ng/mL had about half the risk of SARS-CoV-2 infection, severe COVID-19, or COVID-19 mortality than those not treated [62]. An observational study of 4599 veterans enrolled in U.S. Veterans Affairs health care facilities who had a blood 25OHD test between 20 February and 8 November 2020 had a fully adjusted relative risk of 0.83 (95% CI, 0.72–0.96) for hospitalization for COVID-19 at higher 25OHD concentrations and fully adjusted relative risk of 0.65 (95% CI, 0.50–0.84) for mortality [63].

It is important to highlight that a clinical trial using high-dose cholecalciferol with 95 hospitalized COVID-19 patients in Turkey found a reduction by a factor of 2.14 (95% CI, 1.06–4.33) for mortality rate [64]. A pilot clinical trial in 76 Spanish patients hospitalized for COVID-19 found significantly lower admission to the Intensive Care Unit or mortality for COVID-19 patients treated with high-dose calcifediol shortly after entering the hospital [65]. Subsequent observational studies in Spain found similar results [66]. Thus, there is mounting evidence that higher 25OHD concentrations are associated with reduced risk of COVID-19.

Observational studies can be used to investigate the role of 25OHD concentrations in reducing the risk of SARS-CoV-2 infection and severity of COVID-19. Since having an acute inflammatory illness can lower 25OHD concentrations [67], measurement of 25OHD at the time of COVID-19 diagnosis is not as useful as measurement a few weeks to a few months prior to the disease. However, a recent study from Iran reported that, for 248 COVID-19 patients, 25OHD measured one year before COVID-19 gave similar odds ratios for incidence and death to 25OHD measured at time of admission for COVID-19 [68].

A study from Barcelona reviewed data on prescription supplementation with either cholecalciferol or calcifediol, achieved 25OHD concentrations, and risk of SARS-CoV-2 infection, severe COVID-19, or COVID-19 mortality in comparison with unsupplemented patients with 25OHD concentration <20 ng/mL [62]. Propensity score matching was used to generate appropriate unsupplemented patients. Patients taking cholecalciferol and achieving >30 ng/mL had a significantly lower risk of SARS-CoV-2 infection, severe COVID-19, and COVID-19 mortality. A subsequent article from Andalusia found that the risk of COVID-19 hospitalization was more strongly reduced for cholecalciferol or calcifediol prescriptions 15 days prior to hospitalization than 30 days prior [69].

Another recent article reported findings, from an observational study with U.S. veterans, about the association of vitamin D status and COVID-19-related hospitalization and mortality [63]. The 4599 participants were veterans treated by the U.S. Department of Veteran Affairs health care facilities who tested positive for SARS-CoV-2 and had a vitamin D blood test between 20 February and 8 November 2020, followed for up to 60 days. Vitamin D blood tests were used if obtained between 15 and 90 days prior to testing positive for SARS-CoV-2. Values for many factors were also measured and were used to determine whether they were independent predictors of hospitalization requiring treatment for COVID-19. These factors were included in the models for both hospitalization and mortality concerning 25OHD concentrations. Notably, in going from 15 to 60 ng/mL, hospitalization rates declined from 24.1 to 18.7% (*p* = 0.009), and mortality rates decreased from 10.4 to 5.7% (*p* = 0.001). This observational study appears to be very strong evidence for causality between 25OHD concentrations and severity of COVID-19.

## 4. Long COVID-19 and Vitamin D

The pandemic’s beginning with COVID-19 was characterized by a great concern to contain the contagion of the disease. Almost two years after the health emergency was declared, the focus is the health of those who have survived the disease [70]. As of 18 February 2022, over 409 million confirmed cases and over 5.8 million deaths had been reported globally [71].

These data highlight the large number of people who had COVID-19 and have recovered; in some of them, the consequences will persist in the long term. It is estimated that one-third of patients have persisting symptoms for six months after contracting the infection [72]. Thus, there is an increased need to provide healthcare for long-term symptoms.

The risk factors for long COVID-19 differ somewhat from those for COVID-19 [73]. One study reported that having hypertension, obesity, a psychiatric condition, or an immunosuppressive condition was associated with increased risk of long COVID-19 [74]. On the other hand, long COVID-19 is more likely in women and the age group most affected is somewhat lower. One reason for middle age being a more important risk factor for long COVID-19 is that the risk of mortality increases rapidly with increasing age. A review of COVID-19 mortality rates for 66,646 inpatients in the U.S. admitted from April to June 2020 found increasing mortality rates with increasing age: 40–49 years, 5.8%; 50–59 years, 10.6%; 60–69 years, 18.0%; 70–79 years, 26.5%; and 80+ years, 34.4% [23]. For both COVID-19 mortality and long COVID-19, admission to an intensive care unit is a very important risk factor. Thus, the difference in age profile between mortality and long COVID-19 is that older COVID-19 patients are more likely to die. The reason for more women having long COVID-19 is also likely due to men having a higher mortality rate from COVID-19.

A study involving 4182 COVID-19 cases from Sweden, the UK, and the USA investigated the risk factors for long COVID-19 [75] A total of 558 participants reported symptoms lasting longer than 4 weeks, 189 > 8 weeks, and 95 > 12 weeks. Factors significantly associated with long COVID-19 were age (52 (43–59) years), asthma, heart disease, visit to a hospital, and number of symptoms.

A review of risk factors for long COVID-19 stated that several biomarkers were elevated including D-dimer, interleukin-6 (IL-6), C-reactive protein, procalcitonin, and neutrophils count [76]. A study conducted in western Mexico involving 22 vitamin D supplemented COVID-19 outpatients (mean 25OHD = 22.4 ng/mL) and 20 non-supplemented patients (mean 25OHD = 23.4 ng/mL) found that, although ferritin concentrations were significantly lower in supplemented patients, d-dimer concentrations were not significantly different [33]. A study in India also reported no significant effect on d-dimer concentrations with vitamin D supplementation of 69 COVID-19 patients [77]. Additionally, a high-dose vitamin D supplementation study conducted in Turkey involving 95 hospitalized COVID-19 patients found that increasing mean 25OHD concentration from 23 to 35 ng/mL had no significant effect on ferritin, d-dimer concentrations, but was associated with reduced fibrinogen concentrations [64]. As shown in Table 2, no mechanisms have been shown to reduce concentrations of biomarkers associated with long COVID-19.

Epstein–Barr virus (EBV) reactivation appears to be a risk factor for severe COVID-19 and also appears to be associated with long COVID-19. A study in the UK involving 128 COVID-19 patients, 17 had EBV reactivation and more severe COVID-19 and adverse outcomes [79]. A study in Turkey found long COVID-19 in 56 of 185 COVID-19 patients and that 20 of 30 long COVID-19 patients were positive for EBV reactivation vs. 2 of 20 controls [80]. Vitamin D supplementation of 20,000 IU/week over 96 weeks was found to significantly reduce humoral immune responses to the latent EBV antigen EBNA1 in relapsing-remitting multiple sclerosis [81].

One of the reasons why vitamin D supplementation may be ineffective in treating long COVID-19 is that the SARS-CoV-2 virus can downregulate vitamin D receptors. This has been observed for cytomegalovirus infection [82,83], hepatitis B virus [84], and hepatitis C virus [85]. EBV has been found to block activation of gene expression through is EBNA-3-ptotein [86]. If downregulation is not complete, it might be that very high vitamin D doses would be able to have some effect, but not in the cells with VDRs downregulated.

Although many of the multi-organ manifestations of COVID-19 are known, the possible long-term implications remain unknown [87,88]. Given the recentness of the COVID-19 pandemic, it is not possible to estimate, by itself, the long-term effects. However, there are similar coronavirus events that have happened previously, such as SARS-CoV-1 and Middle East respiratory syndrome coronavirus [89]. Although the current COVID-19 has lower mortality rates than those mentioned above [90], the reports describe similarities, allowing us to know the possible long-term implications and thus take actions to minimize complications [89]. Some of the long-term manifestations of these other pandemics were: matched exercise capacity and carbon monoxide diffusing capacity, cardiovascular complications, hematological manifestations, thrombotic complications, central nervous system manifestations, and renal and gastrointestinal complications [89].

Wang et al. stated that COVID-19 could leave long-lasting consequences in at least three critical areas: pulmonary, neuronal, and neurodegenerative diseases such as Parkinson’s disease, Alzheimer’s disease, and multiple sclerosis [91].

A recent study based on survivors of COVID-19 found that 78 patients out of 100 who had recovered had abnormal cardiovascular findings on magnetic resonance imaging; also, 36 of them suffered dyspnea and unusual fatigue [92]. It should be noted that these consequences were not only observed in those patients who had a severe illness but also in those with mild and moderate presentations [43,93]. Many questions remain unanswered, but the variation in viral load and differential immune response can explain why some have long COVID-19 and others do not [94]. Ahearn-Ford et al. presented data that proposed that inflammatory cytokine pathways altered during infection could continue during convalescence [95].

A recent review stated that the effects on the central nervous system after an acute phase of COVID-19 could be perpetuated over time as a neuro-COVID-19. They reported 12 neurological deficits in long COVID-19, such as mental fog, tremors, confusion, and stiff limbs [96]. For their part, Logue et al. investigated the symptoms that persisted after COVID-19 infection in a longitudinal prospective cohort study with 234 patients. The results show that the most persistent manifestations were fatigue (13.6%) and loss of the sense of smell or taste (13.6%) [72].

The importance of vitamin D in long COVID-19 has recently been published [97]. This pro-hormone, fat-soluble is obtained to a greater extent through sun exposure, but there is also a lower contribution from diet [98]. Diet is an essential determinant of 25OHD concentrations. In particular, Crowe reported that 25OHD concentrations were higher in meat and fish eaters than in vegetarians and vegans, who exclude specific food sources of vitamin D from their diet [1]. More recently, a positive association has been reported between 25OHD concentrations and adherence to the Mediterranean diet, a nutritional pattern effective in preventing and treating obesity-related diseases due to the synergistic action of many nutrients with anti-inflammatory and antioxidant properties [99]. The best-known function of this vitamin is related to the normal mineralization of the bones since it contributes to the absorption of calcium in the intestine and the maintenance of adequate levels of calcium and phosphate in serum, having a fundamental role in the prevention of rickets in children and osteomalacia and osteoporosis in adults [100].

The possibility of vitamin D acting as an immunomodulator has generated great interest recently [101]. However, it has many other functions, including the modulation of cell growth, neuromuscular function, immune function, and a reduction in inflammation [100]. It is important to note that 25OHD concentrations can be decreased in the presence of acute inflammation.

Likewise, more studies are needed to understand better the health impact of the prolonged period of COVID-19 in these patients. Before the pandemic, it was already known that low 25OHD concentrations were associated with fatigue and muscle weakness in the general population. Townsend et al. investigated the relationship between 25OHD concentrations and fatigue and reduced exercise tolerance in 149 patients 79 days after COVID-19 [97]. They evaluated the participants using the Chalder Fatigue score, six-minute walk test, and the modified Borg scale. By applying multivariable linear and logistic regression models, they concluded that there was a correlation between vitamin D and persistent vitamin D fatigue and reduced exercise tolerance in this population of COVID-19 patients. It is important to note that this work only evaluated two of the ample diversity of long COVID-19 symptoms [97]. However, fatigue is the most common symptom of long COVID-19 and is seen in other viral infections [102].

Pizzini et al. studied, in a prospective, multicenter study on long-term sequelae after suffering COVID-19 in 109 patients, the associations of 25OHD concentrations with the presentation of COVID-19 [103]. It was observed that a high proportion of patients presented alteration of vitamin D metabolism eight weeks after diagnosis. Patients with severe COVID-19, most likely due to prolonged hospitalization, showed a disturbing parathyroid-vitamin-D axis within their recovery phase. However, low 25OHD concentrations were not related to the burden of persistent symptoms, concluding that although vitamin D deficiency is common among COVID-19 patients, it was not associated with long-term disease outcomes [103]. Due to the novelty of the disease and the different reported results, it is essential to continue with more studies to evaluate the possible effect of vitamin D in the long post-COVID-19 period.

Overall, the COVID-19 pathology is still characterized by cytokine storm, resulting in endothelial inflammation, microvascular thrombosis, and multiple organ failure [104]. Hyperinflammation is a critical component of severe COVID-19, which is associated with poor outcomes underneath the cytokine storm umbrella term [105]. Thus, an important way to minimize or avoid long COVID-19 is to raise 25OHD concentrations before SARS-CoV-2 infection or COVID-19.

Another way to reduce the risk of long COVID-19 is to aggressively treat SARS-CoV-2 infection and COVID-19 as soon as possible after symptoms are manifest. In one study, raising serum 25OHD concentrations to a mean value near 35 ng/mL in a few days to two weeks for hospitalized COVID-19 patients significantly reduced mortality rates but did not seem to affect symptoms [64]. On the other hand, treating hospitalized COVID-19 patients with high-dose calcifediol has been found to significantly reduce admission to the Intensive Care Unit and death rates [65].

It should be noted that most of the research results are based on COVID-19 variants that are no longer dominant such as the Delta variant. The Omicron variant is associated with 30–45% lower attendance for emergency care and 50–70% lower hospital admission rate in the UK than the Delta variant was [106]. A preliminary report from South Africa also indicates that Omicron COVID-19 is much less severe than Delta COVID-19 [107]. Oxygen therapy use was 75% lower, mechanical ventilation use was nearly 90% lower, admission to intensive care units was about 40% lower, length of stay was 70% lower, and death rates were 90% lower. Although the mean age of Omicron COVID-19 patients (36 years vs. 59 years for Delta COVID-19 patients) explains some of the differences, it implies that those who survived Omicron COVID-19 are much less likely to experience serious long COVID-19. However, it is too soon to determine whether this will be the case.

## 5. Conclusions

The number of COVID-19 diagnosed cases and recovered patients continues to rise with each new SARS-CoV-2 variant, although slowing in summer and fall due to various effects of sunlight. The latter is a particular group that demands special healthcare services since a third of those patients will have persisting symptoms six months or more after recovering from the disease. They will be affected by long-term sequelae causing pulmonary, neuronal, and neurodegenerative diseases.

The functions of vitamin D in bone and calcium metabolism are well-known; however, this pandemic has reinforced the known immunomodulatory effects of this vitamin. Its deficiency has been linked to infectious diseases, some types of cancers, CVD, and chronic inflammation, among others. Additionally, it is related to the severity and mortality of COVID-19 cases, finding a high prevalence of vitamin D deficiency in patients with COVID-19 with acute respiratory failure.

Although more evidence is needed on the effect of vitamin D in COVID-19 (acute and long-term phases), the fundamental role of this vitamin on immune function is evident. So, it appears to be an inexpensive and safe supplement to add as part of COVID-19 treatment.

Given the recent nature of the pandemic and the few studies on prolonged COVID-19 and vitamin D, well-randomized controlled trials are necessary to better understand the role of vitamin D in the protective immune response against prolonged COVID-19. 

## Figures and Tables

**Table 1:** Proposed mechanisms whereby vitamin D reduces risk of COVID-19 (note, order of mechanisms should be carefully considered, perhaps placing more important ones near the beginning).

Effect	Mechanism	Reference
Inactivates viruses	Induction of cathelicidin	[47]
Reduces risk of cytokine storm	Reduces concentration of proinflammatory cytokines and increases concentration of anti-inflammatory cytokines	[24]
Reduces risk of cytokine storm	Induces T regulatory cell production	[27]
Reduces risk of pneumonia	Reduces risk of endothelial dysfunction	[48]
Increases the metabolic tolerance of the host to damage inflicted by the pathogen infection	Reduces matrix metalloproteinase-9 concentrations	[49]
Reduces free SARS-CoV-2 concentrations	Increases soluble ACE2 concentrations that can bind to SARS-CoV-2	[50]
Anti-viral effects	Balanced differentiation of effector CD8 and CD4 T cells	[51]
Reduces risk of myocarditis	Reduces concentration of catecholamines	[52]
Reduces risk of myocarditis	Inhibits RAS	[53]
Reduces risk of vascular dilation and permeability and hypotensin	Inhibits RAS-mediated bradykinin storm	[46]
Protects against the effects of histamines such as acute immune-mediated reactions [54], lung dysregulation [55], increase in Th2 and decrease in Th1 cytokines [56], and thus susceptibility to respiratory tract infections [57]	Preserves stability of mast cells, which can release histamine when activated.	[58]
Promotes adaptive immunity	Regulations of T cell proliferation	[27]
Neuroprotection	Reduces inflammation and oxidative stress	[59]
Protection against exacerbation by other viruses	Reduces risk of Epstein–Barr virus infection	[60]

Abbreviations: SARS-CoV-2 = severe acute respiratory syndrome coronavirus 2; ACE2 = angiotensin-converting enzyme 2; RAS = renin angiotensin system.

**Table 2:** Evidence that vitamin D reduces concentrations of biomarkers associated with long COVID.

Biomarker	Approach	Finding	Reference
D-dimer, a coagulation biomarker	High-dose vitamin D supplementation on COVID-19 patients	No effect	[78]
Procalcitonin	Supplementation with 5000 IU/d vs. 1000 IU/d for 36 and 33 COVID-19 patients	No effect	[77]
Neutrophils count	Supplementation with 5000 IU/d vs. 1000 IU/d for 36 and 33 COVID-19 patients	Significant increase	[77]

Abbreviations: COVID-19 = coronavirus disease 2019; IU = international unit.
